# Investigating the role of Cybersecurity's perceived threats in the adoption of health information systems

**DOI:** 10.1016/j.heliyon.2023.e22947

**Published:** 2023-12-03

**Authors:** Yiyu Zhan, Sayed Fayaz Ahmad, Muhammad Irshad, Muna Al-Razgan, Emad Marous Awwad, Yasser A. Ali, Ahmad Y.A. Bani Ahmad Ayassrah

**Affiliations:** a Civil Engineering College, Putian University, Putian, 351100, China; b Department of Engineering Management, Institute of Business Management, Karachi, Pakistan; c Department of Management Sciences, University of Gwadar, Pakistan; d Department of Software Engineering, College of Computer and Information Sciences, King Saud University, Riyadh, Saudi Arabia; e Electrical Engineering Department, College of Engineering, King Saud University, Riyadh, Saudi Arabia; f Department of Information System, College of Computer and Information Sciences, King Saud University, Riyadh, Saudi Arabia; g Department of Financial and Accounting Science, Middle East University, Amman, 11121, Jordan

**Keywords:** Cyber security threats, Phishing attacks, Ransomware, Lack of skills, Information misuse, Complexity, Vulnerability, Health information system

## Abstract

Information technology is one of the most rapidly growing technologies globally. Over the last decade, its usage in healthcare has been remarkable. Over the last decade, its usage in healthcare has been remarkable. The study examines the impact of various factors as barriers to adopting the information system in healthcare. These factors are categorized into three major types: external attacks, which include phishing attacks and ransomware; employee factors, including lack of skills and the issue of information misuse; and technological factors, including complexity and vulnerability. The findings show that external attacks and technological factors are the main barriers to adopting information systems, while employee factors have no significant impact on the adoption of information systems in the healthcare industry of Pakistan. The study provides implications for healthcare policy makers, professionals and organziations regarding the successful adoption of health information system.

## 1 Introduction

Without a doubt, digitalization or the implementation of advanced information technology plays a vital role in the functioning of modern-day health organizations [1,2]. Yet, it also brings some challenges. One of them is cyber security [3,4]. Protecting information is one of the most critical tasks for health organizations. Most use health information systems like “e-prescribing systems, EHR systems, practice management support systems, clinical decision support systems, radiology information systems, and computerized physician order entry systems,” to name a few. These systems are usually connected to the Internet of Things, the Internet, etc., and have remote access capability [5,6]. It is a fact that such technological systems simplify various tasks and help in the management of healthcare operations. However, the threat it poses to cyber security should not be underestimated [7].

The health sector is an ideal target for cyberattacks due to its apparent cybersecurity flaws. Over the previous three years, about 93 % of medical businesses have suffered a data breach, and 57 % have experienced more than five incidents [8]. Check Point Research says that healthcare organizations worldwide experienced 1463 cyberattacks a week on average in 2022, an increase of 74 % from 2021. Healthcare organizations in the US had 1410 weekly cyberattacks on average, an increase of 86 % from 2021 [9]. It is evident from these statistics that cyberattacks are increasing each year. As more healthcare organizations adopt information systems, more cyberattacks happen. The risk associated with these technologies cannot be ignored and needs proper management steps [10].

On the other hand, the advantages of such technologies can not be ignored in healthcare, and healthcare organizations have no choice but to adopt them [2,11]. Determining the factors behind these attacks in healthcare organizations and how they can be reduced is necessary. Although it is a fact, cybercriminals change their tactics and adopt new attacks according to technology development. The impact of cyberattacks in healthcare is usually large. It may be financial [4,12], personal, or organizational records loss [13], which can be used for many purposes. Adopting HIS is becoming important in the fast growing environment of healthcare to enhance patient care, efficiency in operations, and innovation in healthcare. Regardless of its potential advantages, its adoption varies greatly, with the challenge of perceived cybersecurity risks arising from such systems. Because of the highly sensitive nature of medical information, the healthcare is a prominent target for cyberattacks, and worries regarding breaches of data and privacy, and system weaknesses prevent medical facilities from fully adopting HIS. This study seeks to fill this important gap and to understand the role of perceived cybersecurity threats in HIS adoption, recognizing key factors for improving the security of healthcare system, trust among patients, and overall healthcare.

This research aims to examine the impact of the factors related to cyber security influencing the adoption of the health information system. These factors are categorized into three divisions: external attacks, which include phishing attacks and ransomware; employee factors, including lack of skills and the issue of information misuse; and technological factors, including complexity and vulnerability.

The study has significance since it covers important challenges when integrating healthcare, technology, and security. More than ever, today knowing the role and impact of perceived cybersecurity concerns in the HIS adoption is essential as technology is revolutionising the healthcare. This study offers an understanding regarding how to improve security of patient data, patient trust, healthcare efficiency, provide guidlines for policy and regulation, reduce costs, etc., in the healthcare. Finally, it promises to advance the healthcare industry's capacity to leverage the potential advantages of modern technology whilst protecting the highest levels of patient's security and confidentiality.

## 2 Literature review

### 2.1 Theories

No comprehensive theory is explicitly confined to the risks of using new technologies [14]. However, several frameworks in technology adoption and cyber security address challenges associated with security concerns [15]. The selection of theories is based on their support, relevancy to the study objectives, and ease of integration within a coherent theoretical structure. Although numerous theories related to security and HIS use exist, especially Information Security Management Models and Diffusion of Innovation Theory, their omission was because of limited resources, compatibility with the research objectives, and to keep the investigation focused on its desired scope. These theories can be used in research with a broad scope, variables, recourses, and objectives. Being an important decision in this study, theories selection was determined by their compatibility with research objectives, scope and questions, empirical evidence, and available resources. And these factors were the main influencers of the theory selection process. These frameworks seek to comprehend the variables affecting the adoption of technology as well as how people and organizations could reduce cyber security risks.

The theoretical framework of this study is based on the following three theories.

1. The Protection Motivation Theory (PMT) is a psychological theory that explains how people defend themselves against perceived threats [16]. Understanding how people view cyber security challenges and how their desire to protect from them affects their decision [17]. In the context of this study, people will always consider the risk of perceived threats in the adoption of HIS in healthcare. If they believe it is riskier, they will hesitate to consider the adoption.
2. The Technology Acceptance Model (TAM) emphasizes the perceived usefulness of easy-to-use technology [18]. Users' perceptions of a technology's security and privacy capabilities become crucial when considering cybersecurity concerns [19]. Regardless of the usefulness of technology, people may be reluctant to use it if they perceive it to be insufficiently secure [20]. In the context of this study, people will consider factors like complexity, vulnerability, misuse, and the skills required for it while adopting HIS in healthcare. Even if they believe it is useful, they will consider other factors in making any HIS adoption decisions.
3. The Theory of Planned Behaviour (TPB) concerns how attitudes, subjective norms, perceived behavioral control, and intentions are related [21]. Technology adoption decisions may be influenced by attitudes toward security, subjective norms, and the perception of control over security measures in an environment of cyber security concerns [22]. In the context of this study, the adoption of HIS will also be impacted by individuals' attitudes, norms, and behavior. If they believe cybersecurity concerns a very challenging and important factor, then their decision towards the adoption will be different. Those individuals who perceive cybersecurity and its concerns differently will be more likely to adopt HIS in healthcare.

### 2.2 Phishing

One of the potential challenges the health information system faces is phishing attacks. It is one of the most common methods used by cybercriminals in recent years. It increases daily and poses the greatest potential threat to adopting information systems in the healthcare industry [23]. In such attacks, the attackers impersonate a reliable organization or person through emails or malicious websites to obtain personal information [24]. Once the attackers steal or compromise health information or data, they use it to create duplicate identities, commit insurance fraud, and get medical treatment for free [25].

Hospitals and other healthcare facilities are now using and adopting technologies and software, and the number of attacks is also increasing [26]. Although organizations are also investing in training their employees to use and share information on the technology, etc., cybercriminals are also adopting new ways to commit attacks [27]. Showing resemblance to a trustworthy organization or person, the phishing emails were opened by about 88 % of healthcare workers. In 2021, the types of attacks increased by about 75 %, and criminals invent new methods daily [28]. One of the important reasons for this is that security is not the most prioritized job of healthcare organizations. But now, they are also investing significantly to address the issue and get benefits from the adoption of health information systems [29,30].

Most of today's threat actors still use phishing to exploit their victims. Threat actors continue to develop their tactics, methods, and procedures for the various phishing attacks to maximize the likelihood of successful exploitation [4]. It is one of the most challenging factors for healthcare organizations to implement an information system for healthcare.

### 2.3 Malware

Malware attacks are common cyberattacks that occur when the victim's system is compromised by malware, which is generally malicious software [31]. Ransomware, spyware, command and control, and other targeted attacks are all included in the malicious attack [32]. It is also one of the potential cybersecurity threats for nearly all types of organizations that use the internet and technology. It is always created with corrupt intentions [33]. Malware attacks are increasing against healthcare firms, and according to a survey from cybersecurity company Sophos, two-thirds of healthcare firms were subject to ransomware attacks in 2021, compared to 34 % in 2020 [34]. Health organizations are a popular target for ransomware attacks because they rely so much on access to data, such as patient information, to keep their operations running smoothly. Patients may suffer negative consequences if records are inaccessible promptly [35,36].

Some of the most common objectives of malware attacks are to steal information, payment-related data, credentials, etc., which can sometimes be very costly for healthcare organizations [37]. Such attacks are also used to disrupt routine operations. The level of “disruption” might range from a virus on a single computer damaging crucial operating system data to an organized, physical self-destruction of many systems in an installation. Another possibility involves compromised systems being told to launch extensive distributed denial-of-service assaults. Some malware aims to extract money from the victim directly. Scareware employs fake threats to pressure the victim. Malware, known as “ransomware,” tries to deny a victim access to data until they pay up [38]. In short, malware in any form poses a potential cybersecurity threat to the adoption of healthcare IS. And the number of attacks is increasing day by day.

### 2.4 Data Misuse

Data misuse is using data for a purpose it was not collected for. In the case of misuse, no data breach is involved; there are no hacks of an information system and no ransom, etc. [39]. It comes from the inside of the organization or from its partners with whom the data is shared. Employees, partners, etc., misuse data when they are provided access accidently, use their legal access, and use a shared account [40].

Data Misuse is also one of the human factors linked to the cybersecurity issue in adopting information systems in healthcare. It refers to when a member of the organization steals and uses information for personal gain or corrupt practices [41]. Misuse of data may go unnoticed for a very long period and can severely harm an organization [42]. It is one of the most serious concerns about the use of technology in an organization because many employees have access to the data [43]. Compared to the data of other sectors, the health sector data is relatively more sensitive, so it is misused widely and easily. In a healthcare facility, there are two types of data: patient and research. Both forms of data are equally important and can be misused. For example, a trade secret breach at the Columbus, Ohio-based Research Center of the Nationwide Children's Hospital happened in 2021. The trade secrets of the hospital were sold to China by researchers at the institute, and the ten-year data of various labs were sold to the rivals [44].

In today's cyber world, more health-related data than ever is shared and recorded in the health information system, and the potential threat of misuse exists. There is always the risk of misusing patient and health experiment data. In adopting information systems in healthcare, misuse of information plays a negative role and needs to be addressed.

### 2.5 Human skills

The term “human factors in cybersecurity” describes circumstances in which a security breach is caused by a person making a mistake [45,46]. Humans are the weakest element in an information technology infrastructure and invite a lot of threats. Health organizations are adopting new information technology or information systems, and their employees often struggle to use them properly. New technology requires the latest skills and knowledge, leading to human error or mistakes [47,48].

If an information system is not used properly, it leads to cybersecurity vulnerabilities. According to the “IBM Cyber Security Intelligence Index Report,” 95 % of these data breaches were due to human error or mistake [49]. Misinformation and low skills usually cause human error. Due to these errors, health organizations can face serious economic loss and reputational damage, whether regarding credit card information or other health data [50,51]. In addition, cyber attackers use different means to commit a cyberattack, and the employees have no skills or awareness to recognize them. They share and respond honestly, which leads to cybersecurity issues [52]. Therefore, in adopting information systems in healthcare organizations, the skills of the system users play a significant role. When the organization believes its employees lack sufficient skills, it hinders IS adoption [53].

### 2.6 Complexity

Complexity is also one of the main factors responsible for cybersecurity issues [54] that hinder the adoption of information systems in healthcare [55]. Complexity and security are interrelated, and when any system becomes complex, it becomes more insecure. Complexity often comes with the dependencies of various sub-systems or network elements on each other [56]. The information system often consists of old and new technological devices, which increases security risks [57]. In cyberattacks, the attackers target the complexity of the information system and exploit the data and network. Complex systems are difficult to manage, requiring multi-skilled and advanced-skilled technicians [58]. Not only are those skilled individuals in short supply, but they also do not work for standard pay, making it difficult for the organization to hire or retain them [59].

Information systems are becoming more complex with the advancement of new technologies, and various departments and activities are adopting digital solutions [60,61]. Adopting these technologies compels the organization to rely on digital networks, digital data storage, and other digital devices, which makes the system complex. Usually, a significant part of the system operates outside the firm's environment. It is out of the firm's control, making it hard for the firm to secure or properly manage it [62]. In short, the complexity of technology or information systems has increased cybersecurity risks, and cyberattacks are increasing globally.

### 2.7 Vulnerability

Vulnerability is another important factor that leads to the issue of cybersecurity in the adoption of information systems in healthcare [63]. It is a weakness in a firm's information system and internal control. Cybercriminals target these weaknesses and exploit the system [64]. Many types of vulnerabilities allow attackers to enter the system. Some of the most common examples are that data encryption is lacking, risky files can be uploaded without restriction, code can be downloaded without performing integrity checks, URL redirection to unsafe websites, flawed algorithms, weak passwords, websites without SSL, etc. Cybercriminals use these to enter the system without authorization and exploit the privacy of the data [65].

In this era of information, data is considered a gold mine [66]. It is critical to secure the system from any vulnerability before any compromise of the information system happens [67]. Therefore, minimizing the causes of any information system weakness is important. The system's complexity, device connectivity issues, system management, software bugs, or any other operating system issue could pose a potential threat in the form of a vulnerability [68]. Like the other factors, vulnerability is always challenging for the healthcare information system. Due to its lack of technical skills, low-security budget, and accessibility, healthcare is a soft target for attackers [69]. For the successful adoption of a healthcare information system, vulnerability always plays a negative role, and those who work in healthcare fear that the information system will provide a door for criminals to exploit the health data for their illegal use.

### 2.8 Hypotheses and Conceptual Framework

**Fig. 1:**
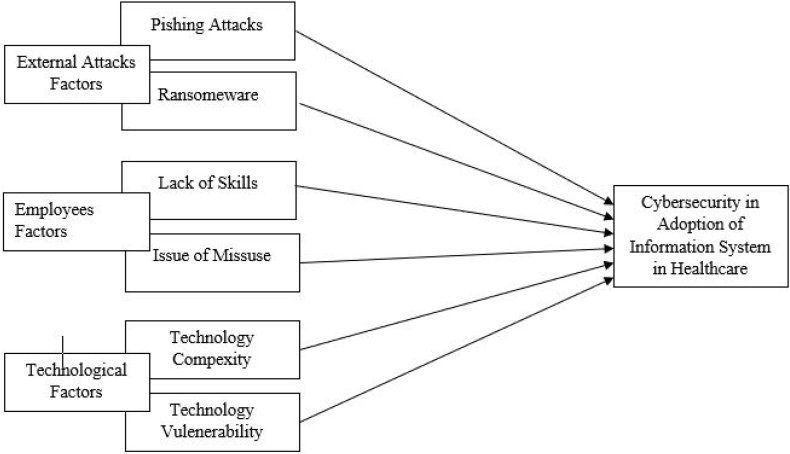
Conceptual framework.

The PMT, TAM, and TPB theoretical frameworks can be used to analyze the effects of technology complexity, vulnerability, misuse, and human skills on adopting HIS in cybersecurity [70]. PMT says that individuals may be discouraged from adopting health information systems due to the perceived threat of complexities and possible vulnerabilities [71,72]. Concerns about technological misuse and people's ability to manage the system safely can further increase people's perception of threat [73]. According to TAM, complexity in HIS may cause resistance among users, particularly healthcare practitioners, due to the difficulties in knowing and securely utilizing the technology [74]. The acceptability may also be hindered by a lack of human skill [75]. According to TPB, negative attitudes due to perceived vulnerability and technological misuse may make people less likely to embrace the HIS [76]. The adoption of HIS is also much influenced by healthcare professionals' trust in their ability to safely manage the system [77]. By considering the above theoretical mechanisms, healthcare organizations can put in place focused strategies to strengthen staff training, foster positive attitudes towards technology adoption, and encourage the safe and successful adoption of HIS in healthcare as shown in Fig. 1. The research concludes the following hypotheses from the literature review in light of the abovementioned theories.

H1 Phishing attacks significantly impact healthcare information systems adoption.

H2 Ransomware significantly impacts the adoption of information systems in healthcare.

H3 Lack of skills significantly impacts healthcare information systems adoption.

H4 The issue of Misuse has a significant impact on the adoption of information systemsin healthcare.

H5 Technology Complexity significantly impacts healthcare information systems adoption.

H6 Technology Vulnerability significantly impacts healthcare information systems adoption.

## 3 Methodology

The philosophy guides the researcher in adopting a methodology for the study. The philosophical foundation of this study is rooted in the positivist paradigm. The positivist paradigm is scientifically based on objective and measurable singular reality. A positivist paradigm is most appropriate as this study is also based on a singular reality based on prior established theories. This study uses a quantitative approach based on deductive reasoning to address the issue in question. Both primary and secondary sources were used for the study. Secondary sources were driven from the prior literature (books, newspapers, etc.) to establish this study's framework. For the analysis and conclusion of this study, primary data were used. Primary data was collected from the respondents through a closed-ended questionnaire. The respondents to this study were limited to medical staff like doctors and nurses in the Pakistani healthcare sector. There are two major types of sampling selection: probability and non-probability sampling [78]. There are basic assumptions for the selection of both; like if the exact number of the research population is known and every individual of the population is accessible by the researcher, then it is better to use probability sampling; other non-probability sampling is recommended if any of the above assumptions is not met. In this research, the exact number of people in the medical field is unknown, so a non-probability sampling technique is recommended according to the direction. Among the several methods of non-probability sampling, purposive sampling is most appropriate for selecting research exact responses. The data was gathered from 506 respondents from Pakistan's medical sector using a purposive sampling technique. A partial least squares structural equation modeling technique via SmartPLS was employed to analyze the primary data. Ethical approval was taken from the Ethics board of the University of Gwadar.

### 3.1 Research instrument

Table 1 shows the details of all the constructs with their respective items and the sources from which they were adapted. Taking care of the validity of the scales, all the scales were adopted from prior valid research. All the measures used for the study were measured on a Likert scale of five points, where 1 represents the least level of agreement, and 5 denotes the highest level of agreement. Research instruments were adopted from previous studies.

**Table 1:** Measurement instrument.

Phishing Attacks [79]	
	1. I always received fake links about healthcare facilities.
	2. There are several fake sites that resemble healthcare sites.
	3. I always received fake emails regarding healthcare services.
Ransomware [80]	
	1. I have faced viral attacks during online surfing.
	2. Several times, I have lost my documents due to viral attacks.
	3. Due to viral attacks, I was unable to access my documents.
Lack of Skills [81]	
	1. Human soft skills are very necessary for using modern IT-based technology.
	2. Developed IT skills will motivate a person to use IT services.
	3. A person with IT skills will better use the IT services.
	4. I am skillful in using Healthcare information systems.
	5. Lack of IT skills is a barrier to using healthcare information systems.
Issue of Misuse [82]	
	1. I don't feel sharing my personal information on internet-based software is easy.
	2. I have faced information misuse problems with IT-based devices.
	3. Personal information is not safe in the healthcare information system.
	4. People mostly misuse the information obtained from the internet.
Technology Complexity [83]	
	1. People prefer to use easy-to-use technology.
	2. People avoid health information systems due to their complexity.
	3. People hesitate to adopt modern technologies due to their complexity.
Technology Vulnerability [84]	
	1. I consider that modern technology is not safe for the user.
	2. There are several chances for online attacks on IT-based systems.
	3. The risk of information theft on IT-based systems is increasing day by day.
Adoption of an Information System [85]	
	1. Information system pulls together services that are offered by different areas in the hospitals.
	2. Information systems effectively integrate services from different areas of the hospitals.
	3. The information system enables me to access services from anywhere within the hospitals (dropped)
	4. The information system provides data that is accurate.
	5. The information system provides data that is well formatted.
	6. The information system provides real-time data (dropped) information system provides real-time data (dropped)
	7. Using the information system improves my effectiveness.
	8. Using the Information system improves my performance.
	9. Using the information system enhances my productivity.
	10. Overall, using an information system is useful.

### 3.2 General characteristics of the sample

Table 2 shows the general characteristics of the study sample. The table shows 506 respondents, of whom 49.4 % are male and 50.6 % are female. The second section of the table indicates the age-wise distribution of the respondents. This section shows that there are a total of four age groups. The highest percentage of the respondents were between the age group of 20–30 years, with a percentage of 39.5 %, while the lowest were 51 and above years, with a percentage of 7.1 %. The table's third and last section indicates the designation-wise distribution of the respondents. This section denotes a total of 49.8 % doctors and 50.2 % nurses.

**Table 2:** Sample characteristics.

Gender	Number	Percentage
Male	250	49.4 %
Female	256	50.6 %
Total	506	100.0 %
**Age Group**	**Number**	**Percentage**
20–30 Years	200	39.5 %
31–40 Years	159	31.4 %
41–50 Years	111	21.9 %
51 and above Years	36	7.1 %
Total	506	100.0 %
**Designation**	**Number**	**Percentage**
Doctor	252	49.8 %
Nurse	254	50.2 %
Total	506	100.0 %

### 3.3 Frequency of the respondents

Table 3 of the respondents' frequency denotes the percentage of the respondents who have rated each question.

**Table 3:** Frequency of respondents.

Construct	Items	Strongly Disagree	Disagree	Neutral	Agree	Strongly Agree
**Phishing Attacks**	PA1	5 %	3 %	17 %	52 %	24 %
	PA2	1 %	7 %	15 %	52 %	26 %
	PA3	3 %	8 %	12 %	53 %	24 %
**Ransomware**	RW1	3 %	9 %	18 %	57 %	12 %
	RW2	4 %	8 %	16 %	58 %	14 %
	RW3	5 %	10 %	25 %	50 %	10 %
**Lack of Skills**	LS1	4 %	9 %	18 %	58 %	11 %
	LS2	4 %	9 %	20 %	57 %	10 %
	LS3	4 %	10 %	13 %	58 %	15 %
	LS4	4 %	13 %	26 %	41 %	16 %
	LS5	4 %	26 %	4 %	25 %	41 %
**Issue of misuse**	IM1	7 %	18 %	7 %	20 %	48 %
	IM2	12 %	27 %	5 %	21 %	34 %
	IM3	10 %	20 %	8 %	21 %	41 %
	IM4	12 %	23 %	15 %	26 %	23 %
**Technology Complexity**	TC1	5 %	4 %	14 %	45 %	32 %
	TC2	6 %	5 %	22 %	42 %	25 %
	TC3	4 %	10 %	23 %	41 %	21 %
**Technology Vulnerability**	TV1	4 %	8 %	15 %	51 %	22 %
	TV2	25 %	21 %	28 %	10 %	16 %
	TV3	17 %	7 %	7 %	9 %	61 %
**Adoption of an Information System**	AS1	6 %	13 %	14 %	32 %	35 %
	AS2	2 %	3 %	14 %	26 %	56 %
	AS3	3 %	9 %	25 %	27 %	36 %
	AS4	2 %	5 %	14 %	58 %	22 %
	AS5	2 %	4 %	15 %	55 %	24 %
	AS6	1 %	7 %	10 %	59 %	24 %

## 4 Results

### 4.1 Reliability and convergent validity

Reliability is the measure used to determine the degree to which the results of a measurement can be stable and consistent. Two types of common reliability are used in structural equation modeling: item reliability and construct reliability [86]. The measure used for the items' reliability is their outer loading values. The ideal threshold value for the outer loading is 0.7, but in many cases, even a value greater than 0.5 is also acceptable if the initial criteria of the construct's overall reliability is established [87]. Table 4 of the reliability and convergent validity shows that all the outer loading values are greater than the minimum threshold value, indicating that all the model items have achieved their reliability. The measure used for construct reliability is Cronbach's alpha and composite reliability. The threshold value for both measures is 0.7. Table 4 of reliability and convergent validity shows that all the reliability values are within the threshold range, which indicates that all the constructs are reliable. Validity is the extent to which the instruments used in the research measure exactly what you want them to measure [88]. There are two types of validity scales: convergent and discriminant validity. The average variance extracted (AVE) is the convergent validity measure. The ideal threshold value for the AVE is 0.5 and above. Still, a value of 0.4 is also acceptable if the initial reliability criteria for the items and constructs are established [89]. Table 4 shows that all the AVE values are above the threshold, indicating that all the constructs are convergently valid. During the confirmatory factor analysis, four items of adoption of IS were removed from the model due to insignificant outer loading values, while the rest of the other measures were significant and are part of the model as it is.

**Table 4:** Reliability, multicollinearity and convergent validity.

Construct	Items	Outer loading	VIF	CA	CR	AVE
Adoption of IS	AS1	0.738	1.581	0.738	0.82	0.436
	AS2	0.798	1.721			
	AS3	0.598	1.23			
	AS4	0.535	1.251			
	AS5	0.609	1.296			
	AS6	0.647	1.388			
Issue of Misuse	IM1	0.827	1.951	0.825	0.884	0.656
	IM2	0.758	1.494			
	IM3	0.815	1.890			
	IM4	0.837	1.783			
Lack of Skills	LS1	0.828	2.074	0.867	0.904	0.653
	LS2	0.857	2.377			
	LS3	0.792	1.808			
	LS4	0.788	1.819			
	LS5	0.773	1.777			
Phishing Attacks	PA1	0.851	1.816	0.82	0.893	0.736
	PA2	0.860	1.890			
	PA3	0.862	1.800			
Ransomware	RW1	0.866	1.910	0.871	0.921	0.795
	RW2	0.902	2.743			
	RW3	0.907	2.832			
Technology Complexity	TC1	0.699	1.239	0.697	0.832	0.624
	TC2	0.805	1.451			
	TC3	0.857	1.573			
Technology Vulnerability	TV1	0.815	1.63	0.76	0.861	0.674
	TV2	0.826	1.629			
	TV3	0.823	1.420			

### 4.2 Discriminant validity

Discriminant validity is the measure used to detect how much one construct differs from another. Three common measures used for discriminant validity are named Fornell-Larcker criteria, Heterotrait-Monotrait (HTMT) values, and cross-loadings.

#### 4.2.1 Fornell Larcker Criteria

The first criterion for discriminant validity is the Fornell Larcker Criteria. The threshold value for the Fornell-Larcker criteria is that all the diagonal values must be greater than the respective values of their columns and rows, as shown in Table 5.

**Table 5:** Fornell Larcker criteria.

	Adoption of IS	Issue of Misuse	Lack of Skills	Phishing Attacks	Ransomware	Technology Complexity	Technology Vulnerability
Adoption of IS	**0.866**						
Issue of Misuse	0.512	**0.810**					
Lack of Skills	0.621	0.413	**0.808**				
Phishing Attacks	0.703	0.418	0.642	**0.858**			
Ransomware	0.719	0.438	0.505	0.507	**0.892**		
Technology Complexity	0.766	0.590	0.606	0.608	0.767	**0.790**	
Technology Vulnerability	0.702	0.366	0.630	0.585	0.575	0.553	**0.821**

#### 4.2.2 Heterotrait-Monotrait (HTMT) ratios

The second measure used for discriminant validity is HTMT values. The threshold value for the HTMT value is 0.85 or less. Table 6 shows that all the HTMT values are smaller than the threshold value, which indicates that all the constructs are discriminately valid.

**Table 6:** HTMT.

	Adoption of IS	Issue of Misuse	Lack of Skills	Phishing Attacks	Ransomware	Technology Complexity
Issue of Misuse	0.638					
Lack of Skills	0.746	0.491				
Phishing Attacks	0.803	0.507	0.754			
Ransomware	0.808	0.512	0.576	0.596		
Technology Complexity	0.809	0.773	0.769	0.707	0.773	
Technology Vulnerability	0.816	0.458	0.778	0.737	0.696	0.747

#### 4.2.3 Cross-loading

The third measure used for discriminant validity is cross-loading. The threshold value for the cross-loading is that the self-loading of the construct is greater than the cross-loading. Table 7 shows that all the self-loading values are greater than the cross-loading values, indicating that all the constructs are discriminately valid.

**Table 7:** Cross-loading.

	Adoption of IS	Issue of Misuse	Lack of Skills	Phishing Attacks	Ransomware	Technology Complexity	Technology Vulnerability
AS1	**0.738**	0.328	0.485	0.628	0.505	0.500	0.561
AS2	**0.798**	0.485	0.574	0.610	0.495	0.583	0.694
AS3	**0.598**	0.282	0.463	0.481	0.391	0.423	0.545
AS4	**0.535**	0.230	0.219	0.290	0.451	0.415	0.248
AS5	**0.609**	0.362	0.287	0.347	0.467	0.571	0.286
AS6	**0.647**	0.296	0.335	0.328	0.568	0.550	0.313
IM1	0.383	**0.827**	0.359	0.373	0.307	0.442	0.217
IM2	0.404	**0.758**	0.373	0.327	0.374	0.471	0.442
IM3	0.375	**0.815**	0.314	0.295	0.346	0.485	0.276
IM4	0.481	**0.837**	0.300	0.355	0.385	0.507	0.253
LS1	0.542	0.396	**0.828**	0.601	0.468	0.558	0.531
LS2	0.522	0.278	**0.857**	0.534	0.459	0.563	0.516
LS3	0.497	0.335	**0.792**	0.525	0.382	0.447	0.516
LS4	0.495	0.383	**0.788**	0.500	0.398	0.500	0.431
LS5	0.445	0.272	**0.773**	0.417	0.321	0.357	0.558
PA1	0.587	0.338	0.525	**0.851**	0.498	0.514	0.491
PA2	0.586	0.411	0.483	**0.86**	0.385	0.528	0.452
PA3	0.633	0.329	0.636	**0.862**	0.423	0.521	0.558
RW1	0.665	0.458	0.523	0.578	**0.866**	0.713	0.516
RW2	0.629	0.357	0.418	0.386	**0.902**	0.645	0.544
RW3	0.626	0.353	0.406	0.384	**0.907**	0.691	0.478
TC1	0.513	0.375	0.434	0.471	0.427	**0.699**	0.38
TC2	0.614	0.543	0.412	0.463	0.743	**0.805**	0.402
TC3	0.676	0.473	0.579	0.510	0.627	**0.857**	0.520
TV1	0.519	0.286	0.554	0.510	0.354	0.392	**0.815**
TV2	0.556	0.258	0.465	0.430	0.494	0.439	**0.826**
TV3	0.641	0.349	0.534	0.500	0.550	0.519	**0.823**

### 4.3 Common method bias

Common method bias is a spurious variance that attributes the measurement method rather than the construct the measures are assumed to represent. It is a major issue faced by the researcher working with primary data. Variance Inflation Factor (VIF) values reflect the multicollinearity issue of the model and address the common method bias problem. A model with VIF values less than 3.0 indicates that it is free from common method bias. Table 3 of the reliability, multicollinearity, and convergent validity shows that all the items of the individual construct have a VIF value less than the threshold value, indicating that the model is free from the issue of common method bias.

### 4.4 Model fitness

Once the reliability and validity of the measurement model are confirmed, the structural model's fitness must be assessed in the next step. For the model fitness, several measures are available in the SmartPLS, like the SRMR, Chi-square, NFI, etc., but most of the researchers recommend the SRMR for the model fitness in the PLS-SEM. A value less than 0.08 is generally considered a good fit when applying PLS-SEM. However, Table 8 shows that the SRMR value is 0.071, less than the threshold value of 0.08, indicating that the model is fit.

**Table 8:** Model fitness.

	Saturated Model	Estimated Model
SRMR	0.071	0.071
d_ULS	2.463	2.463
d_G	0.892	0.892
Chi-Square	2482.729	2482.729
NFI	0.703	0.703

### 4.5 Regression analysis and hypothesis testing

Table 9 shows the regression analysis of the study. It indicates that there is a total of six relationships based on the hypothesis of this study. All the hypotheses are based on a direct relationship. The findings support four hypotheses, as shown in the following interpretations.

**Table 9:** Path coefficient.

Hypothesis	β	T Statistics	P Values	Results
H1: Phishing Attacks - > Adoption of IS	0.255	6.257	0.000	*Supported*
H2: Ransomware - > Adoption of IS	0.199	4.216	0.000	*Supported*
H3: Lack of Skills - > Adoption of IS	0.003	0.091	0.928	*Not Supported*
H4: Issue of Misuse - > Adoption of IS	0.057	1.865	0.063	*Not Supported*
H5: Technology Complexity - > Adoption of IS	0.280	5.748	0.000	*Supported*
H6: Technology Vulnerability - > Adoption of IS	0.265	6.397	0.000	*Supported*

H1 (supported): The results show a positive and significant relationship between phishing attacks and the adoption of 10.13039/100015147IS, with a coefficient of 0.255, a t-value of 6.257, and a p-value of 0.000. This shows that phishing attacks influence the IS's adoption decisions. In other words, decision-makers will consider the potential of an IS to face and address phishing attacks.

H2 (supported): The results also show a positive and significant relationship between ransomware and the adoption of 10.13039/100015147IS, with a coefficient of 0.199, a t-value of 4.216, and a p-value of 0.000. This shows that ransomware also influences the IS's adoption decisions. In other words, decision-makers will consider the potential of an IS to face and address the ransomware.

H3 (not supported): The results show no significant relationship between the lack of skills and the adoption of 10.13039/100015147IS, with a coefficient of 0.003, a t-value of 0.091, and a p-value of 0.928. The findings show that employees' skills have no impact on HIS adoption. It is important to highlight that adopting new technology requires skills, but the employees may not necessarily possess them before adopting technology. These skills may be obtained through on-the-job training. Therefore, the lack of skills has shown no significant relationship with the adoption of HIS.

H4 (not supported): The results also show no significant relationship between misuse and the adoption of 10.13039/100015147IS, with a coefficient of 0.057, a t-value of 1.865, and a p-value of 0.063. Any technology can be misused but cannot influence the organization's adoption decisions. Especially, the fear of issue does not influence the adoption of HIS. This also shows the confidence and trust of the employees in themselves and the organization.

H5 (supported): The results also show a positive and significant relationship between technology complexity and the adoption of 10.13039/100015147IS, with a coefficient of 0.280, a t-value of 5.748, and a p-value of 0.000. Technology complexity has an impact on the adoption of HIS. Ease of use is one of the main aspects of the technology acceptance model. If any technology is complex, it won't be easy to use and, therefore, will be seldom adopted.

H6 (supported): The results also show a positive and significant relationship between technology vulnerability and the adoption of 10.13039/100015147IS, with a coefficient of 0.265, a t-value of 6.397, and a p-value of 0.000. It is evident from the values that the vulnerability of any technology is linked with its adoption. Organizations and individuals consider this aspect of technology while adopting new technology. In healthcare, the decision regarding the adoption of HIS is impacted by the level of technology vulnerability.

### 4.6 Structural model

**Fig. 2:**
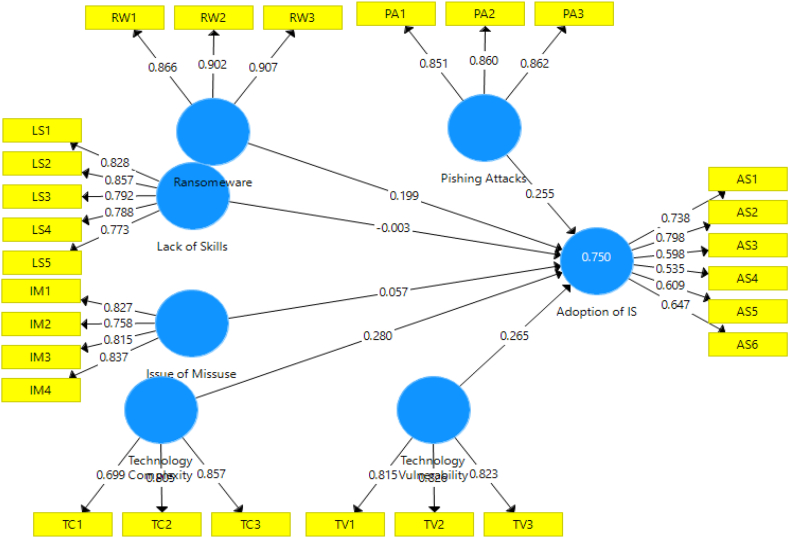
Tested Structural Model

Fig. 2 shows the relationships among the variables used in the model.

### 4.7 Coefficient of determination

The coefficient of determination explains the rate of the percentage of variation in the dependent variable by the effect of the collective independent variables. The measure used for the coefficient of determination is R square. Table 10 shows that the R square value is 0.750, which indicates that the study's independent variables cause 75 % of the variation in the dependent variable.

**Table 10:** Of R square.

	R Square	R Square Adjusted
Adoption of IS	0.750	0.747

### 4.8 Predictive relevance of the model

The predictive relevance of the model is an advanced technique used in partial least squares to explain the prediction power of the model of the study and how much the same model will predict in a different situation. The measure used for predictive relevance is Q square. According to the statistician and the researcher, a value above zero is considered a good prediction. Table 11 shows that the Q square value is 0.315, which explains the model's good predictive power.

**Table 11:** Of Q square.

	SSO	SSE	Q^2^ (=1-SSE/SSO)
Adoption of IS	3036	2079.157	0.315
Issue of Misuse	2024	2024	
Lack of Skills	2530	2530	
Phishing Attacks	1518	1518	
Ransomware	1518	1518	
Technology Complexity	1518	1518	
Technology Vulnerability	1518	1518	

### 4.9 IPMA analysis

**Fig. 3:**
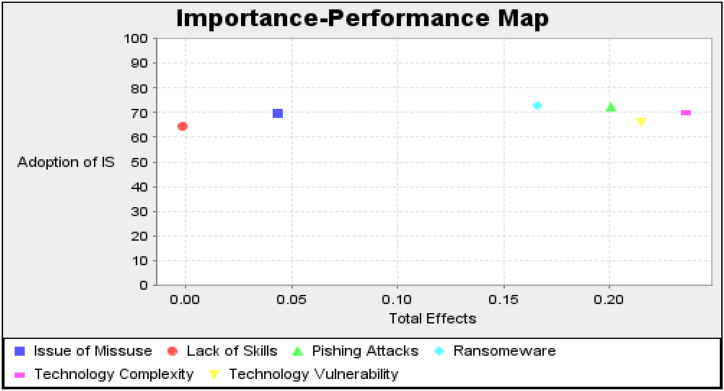
Impotance-Performance Map

IPMA analysis is an advanced technique used in the SmartPLS to explain the importance and performance of the individual variables for the study's main variable, the adoption of the information system. Table 12 shows that technology complexity is the highest importance variable, with a value of 23.6 %. However, there is still a need to increase the performance to the maximum level of 100 %, which is currently 70.021 %. Fig. 3 indicates the graphical representation of the variables' importance and performance.

**Table 12:** Of IPMA analysis.

	Importance	Performances
Issue of Misuse	0.043	69.683
Lack of Skills	−0.002	64.39
Phishing Attacks	0.201	72.276
Ransomware	0.166	73.021
Technology Complexity	0.236	70.021
Technology Vulnerability	0.215	66.061

### 4.10 MGA analysis

MGA stands for Multi Group Analysis, an advanced technique used in the SmartPLS to compare the effect of the relationship based on the two groups of people. Table 12 shows the gender-based difference, which explains the difference between males and females. The measure used for the significant effect is the p-value. The threshold value for the p-value is 0.05 or less. Table 13 shows that there is no significant impact due to gender.

**Table 13:** Of MGA analysis based on gender.

	β-diff (Male-Female)	p-Value (Male-Female)
Issue of Misuse - > Adoption of IS	−0.012	0.832
Lack of Skills - > Adoption of IS	−0.034	0.645
Phishing Attacks - > Adoption of IS	0.049	0.517
Ransomware - > Adoption of IS	0.056	0.531
Technology Complexity - > Adoption of IS	−0.118	0.229
Technology Vulnerability - > Adoption of IS	0.017	0.825

Table 14 shows the designation-based difference that explains the difference between doctors and nurses. The measure used for the significant effect is the p-value. The threshold value for the p-value is 0.05 or less. Table 14 shows that there is no significant impact due to designation.

**Table 14:** Of MGA analysis based on designation.

	β-diff (Doctor-Nurse)	p-Value (Doctor-Nurse)
Issue of Misuse - > Adoption of IS	−0.038	0.514
Lack of Skills - > Adoption of IS	0.014	0.851
Phishing Attacks - > Adoption of IS	0.082	0.270
Ransomware - > Adoption of IS	0.07	0.507
Technology Complexity - > Adoption of IS	−0.052	0.622
Technology Vulnerability - > Adoption of IS	−0.106	0.200

## 5 Discussion

The study examines different factors related to cyber security threats and their impact on adopting the information system in healthcare. These factors are categorized into three types: external attack, employee, and technological factors. External attack factors are phishing attacks [90]. and ransomware [91], employee factors are lack of skills [92] and issues of misuse [93]; and technological factors are complexity [94] and vulnerability [95]. According to the literature, several factors are responsible for adopting the healthcare information system in the healthcare sector. Still, in the context of Pakistan, these are the three major factors responsible for contributing.

The first hypothesis says that phishing attacks significantly impact the adoption of the information system in the healthcare sector of Pakistan. The findings of this study also support this relationship, with β = 0.25 and p = 0.00. The past literature on adopting information systems in healthcare or any other sector shows different cyber factors, but phishing attacks are among the most dominant viral attacks [96]. The second hypothesis says that ransomware is a type of cyberattack that significantly impacts the adoption of information systems in the healthcare sector of Pakistan. The findings of this study also support the relationship with β = 0.199 and p = 0.000. Many other researchers have found the same results for ransomware as a barrier to adopting the information system in the healthcare industry and other sectors like hospitality in different contexts [97]. The third hypothesis, “Lack of skills significantly impacts the adoption of information systems in healthcare,” was not supported by the statistical analysis with β = 0.003 and p = 0.928. Literature is divided about this. Some papers support these results, and some do not [90]. The main reason for these inconsistent findings may be the human mentality of those adopting the system [98].

The fourth hypothesis is that " misuse significantly impacts adopting information systems in healthcare.” However, the findings don't support the argument that the information misuse issue is not a sufficient factor that should be responsible for the design problem, with β = 0.057 and p = 0.063. Other studies also show inconsistent results, although, in some cases, they have a significant impact. In some cases, it doesn't show a significant impact. These inconsistent findings in the study and the literature may be because most users are unaware of how a user's personal information can be misused. They have high confidence in these systems and no cybersecurity concerns [99]. The fifth hypothesis, “Technology Complexity Significantly Impacts the Adoption of Information Systems in Healthcare,” is supported by the results. Like other factors, technology complexity is one of the major factors that will be a barrier for the user to adopt the healthcare information system in the Pakistani context, with β = 0.280 and p = 0.000. Many other studies have also found the same results [100,101]. The sixth hypothesis says that technology vulnerability may be a sufficient factor to act as a barrier for the users to adopt the healthcare information system in Pakistan. The findings of this study also support the notion that technology vulnerability is one of the main factors responsible for the said issue, with β = 0.265 and p = 0.000. The past literature based on the same factor but tested in a different context and different industry also found the same result [102,103].

In summary, while the assumptions regarding skill deficiencies and misuse are not verified, those regarding phishing attacks, ransomware, technology complexity, and vulnerability are. The strong positive correlation between the adoption of IS and phishing attacks, ransomware, technological complexity, and technology vulnerability implies that these security concerns are the key obstacles to IS adoption in healthcare organizations and must be eliminated. Ransomware and phishing attacks can seriously harm an organization's finances and reputation and threaten healthcare organizations' adoption of IS. Healthcare organizations must detect, mitigate, and prevent these risks. Similarly, technical vulnerability and complexity might raise the possibility of security breaches, which could endanger the uptake of IS. Therefore, the IT infrastructure needs to be simple and secure. i.e., the IS needs to be useable by the employees and less vulnerable to attack. A lack of skills not significantly correlated with IS adoption raises the possibility that IS adoption may not be at risk from cyberattacks due to a shortage of skills. This result is surprising as individuals and organizations struggle with a skills gap. However, it's feasible that healthcare firms use other tactics like outsourcing to overcome the skills gap. The weak positive correlation, which is not statistically significant, between the misuse problem and IS adoption suggests that technology misuse may not necessarily be a reason for cybersecurity threats in adopting IS in healthcare. This result may be due to the possibility that detecting misuse using IS alone may be challenging and may instead call for other technical and non-technical approaches. These results have implications for healthcare organizations seeking to adopt IS. Organizations must consider the security risks they confront to reduce cybersecurity threats to guarantee a successful adoption of IS. When adopting IS, organizations should take organizational considerations into account in addition to technical ones.

## 6 Conclusion

This study examines the impact of the factors that threaten the users' adoption of the healthcare information system. Literature has found several factors that may act as barriers to the said issue. According to the literature, these factors are divided into three categories: external attacks, employee factors, and technology-based factors. External attacks include phishing attacks and ransomware; employee factors include a lack of skills and information misuse issues; technological factors include complexity and vulnerability. From the results of this study, it was found that external attacks and technological factors have a significant impact on acting as a barrier to the adoption of the information system in the healthcare industry, while employee or personal factors, such as misuse or a lack of skills, have no significant impact on acting as a barrier to the adoption of the information system in healthcare.

### 6.1 Theoretical and managerial implication

#### 6.1.1 Theoretical implications

The study indicates that the implementation of IS is subject to a wide range of interconnected cybersecurity concerns. The significant and positive correlation between ransomware, phishing attacks, technology complexity, and technology vulnerability emphasizes the importance of knowing cyber threats' dynamic and complex nature. The study also stresses addressing specific dangers and the fundamental causes of their creation and spread. The influence of cyber risks on the adoption of IS can be prevented and mitigated by organizations by recognizing these aspects.

##### 6.1.1.1 Managerial implications

These results show how critical it is to establish an integrated and proactive strategy for cyber security when adopting IS. Organizations can more effectively protect their information technology systems by addressing the root causes of cyber threats at the technology, organizational, and user levels.

1. Organizations must prioritize investments in cyber security measures to combat the wide range of cyber threats. This entails creating strong security protocols and systems, spending money on programs that train and make employees aware of security issues, and constantly monitoring and improving security measures to keep up with new threats.
2. Organizations must take action to lessen the complexity and vulnerability of technology. This can involve simplifying the infrastructure of IT, routine hardware and software updates, and prompt installation of security fixes and updates. Organizations should also put in place mechanisms to identify and address vulnerabilities.
3. Organizations must implement preventative measures to deal with ransomware and phishing assaults. This involves creating and testing incident response strategies, installing anti-phishing and anti-ransomware technology, and training staff members on recognizing and preventing phishing attempts and other forms of social engineering.

### 6.2 Limitations and future research direction

• This study was based on a quantitative approach, relying only on the factors already found by the researchers in different contexts. Further researchers can use a qualitative approach to explore new factors that may be a barrier to adopting the information system in healthcare.
• Many essential aspects of technology related to the adoption, like integration, interoperability, communication, supplier security practices, resource constraints, etc., can be considered in future studies, which were not included in this study due to its limited scope.
• The scope of this study is limited to Pakistan; further researchers can test the model in different geographical contexts and industries.

## Data availability statement

Data will be available on request from the corresponding author.

## Funding

The study is supported by Researchers Supporting Program number (RSP2023R206), 10.13039/501100002383King Saud University, Riyadh, Saudi Arabia.

## CRediT authorship contribution statement

**Yiyu Zhan:** Conceptualization. **Sayed Fayaz Ahmad:** Writing – original draft, Conceptualization, Supervision. **Muhammad Irshad:** Data curation. **Muna Al-Razgan:** Formal analysis, Funding acquisition. **Emad Marous Awwad:** Validation. **Yasser A. Ali:** Methodology, Writing – review & editing. **Ahmad Y.A. Bani Ahmad Ayassrah:** Methodology, Writing – review & editing.

## Declaration of competing interest

The authors declare that they have no known competing financial interests or personal relationships that could have appeared to influence the work reported in this paper.
